# Incidence and potential causative factors associated with chronic benign neutropenia in the Kingdom of Saudi Arabia

**DOI:** 10.1186/1753-6561-9-S2-S1

**Published:** 2015-01-15

**Authors:** Mamdooh Gari, Mohammed Dakhakhni, Abdullah Gari, Erada Alshihri, Rowan Al-Jahdali, Kothandaraman Narasimhan, Shen Liang, Fatin Al-Sayes, Gauthaman Kalamegam, Adeel Chaudhary, Adel Abuzenadah, Mohammed Al-Qahtani

**Affiliations:** 1 Department of Medical Laboratory Technology, Faculty of Applied Medical Sciences, Research, King Abdulaziz University PO Box 80216, Jeddah 21589, Saudi Arabia; 2 Centre for Excellence in Genomic Medicine Research, King Abdulaziz University PO Box 80216, Jeddah 21589, Saudi Arabia; 3 Faculty of Medicine, Department of Hematology, King Abdulaziz University Hospital, Jeddah, Saudi Arabia; 4 Biostatistics Unit at the Yong Loo Lin School of Medicine, National University of Singapore, Singapore

## Abstract

**Background:**

Benign neutropenia often presents in certain populations without any genotype nor phenotype. Middle East countries are among the regions where endemic cases of chronic benign neutropenia are reported in the general population with an incidence of approximately between 10-15%. Not many studies have been performed to ascertain the cause or burden associated with this condition. The objective of the current study was to identify the frequency and characterize the consequences of chronic benign neutropenia in the country of Saudi Arabia.

**Results:**

Benign neutropenia was found to be high in the Saudi Arabia general population (up to 20%), with an average neutrophil count of 1.48 (range 0.99 – 1.95 × 10^9^cells/L), with Saudis having a higher incidence of chronic benign neutropenia compared to non-Saudis (p = <0.05). Complete blood count analyses showed significant difference in the total white cell count of neutrophils (p < 0.0001), WBC (p < 0.0001), lymphocytes (p < 0.001), monocytes (p < 0.001), eosinophils (p = 0.013) as well as the CD19 B cells (p = 0.008).

**Conclusions:**

Our study is the first to carefully quantitate benign neutropenia in Saudi Arabia. We identified that this condition is prevalent in the middle aged population (18 years to 55 years). These individuals not only had lower neutrophil counts, but also reduced peripheral blood cells types, especially the B-lymphocyte population (CD19 subset). As B-lymphocytes are involved in antibody production and antigen recognition, a decrease might easily predispose the individuals to infectious agents. As such more mechanistic studies need to be undertaken to understand the cause and potential long-term consequences of benign neutropenia.

## Background

Neutropenia is commonly defined as a decrease in the absolute number of neutrophils, which is the predominant cell type among the various fractions of the white blood cells. Abnormal low levels of neutrophils (neutropenia) is an important risk factor for infection, and may be classified as mild (1.0 to 1.5 x 10^9^/L), moderate (0.5 to 1.0 x 10^9^/L), or severe (<0.2 x 10^9^/L, 2 < 0.5 x 10^9^/L) [1]. If left untreated this could lead to different forms of chronic disease conditions [2]. Neutropenia is broadly classified into the severe chronic neutropenia (SCN) and acquired or secondary neutropenia [3]. SCN includes the heterogeneous group of congenital conditions (bone-marrow failure syndromes, inborn errors of metabolism, immunodeficiencies etc); as well as cyclical and idiopathic neutropenia [3]. Acquired neutropenia can be due to drug toxicity, radiotherapy, infection, immune mediated diseases, haematological diseases and collagen vascular disorders [3]. However, neutropenia is mostly associated with patients undergoing chemotherapy or radiotherapy for malignancies such as leukaemia, lymphoma and thymoma. Benign ethnic neutropenia is another form of neutropenia and is prevalent in approximately 25% to 50% of the African as well as Middle Eastern population [1]. Individuals with benign neutropenic condition usually present with consistent low absolute neutrophil counts with no obvious increased susceptibility to infection.

It has been observed that certain healthy people at routine checkups present with low neutrophil counts that is not associated with any obvious disease burden [4,5]. This condition is known as benign neutropenia or in some cases chronic benign neutropenia (CBN). Chronic benign neutropenia in otherwise healthy subjects has been observed in different groups, such as Africans [6], African Caribbean people [7], Ethiopians, Yemenite Jews [8,9] and certain Arab populations. Prevalence of isolated endemic incidences of benign neutropenia has been previously reported in the Middle Eastern countries [8,10,11]. However, the specific risk of defined genetic subgroups has largely remained unknown. A recent report by Denic et al., (2010) [4] showed that the mean neutrophil count in Arabs is lower than those found in individuals who are from European origin [4]. The root cause for the origin of this benign condition is still not understood. Understanding the cause for chronic benign neutropenia (CBN) will be crucial for the management of this disease in the general population. A genetic cause, an autosomal dominant mode for transmission of the disease [4], has been postulated however this requires extensive validation studies.

The incidence of CBN condition shows variation between males and females with males being more prone to this condition [5]. Recurrent infections, especially chest infection, bacterial and viral infections are some indicators for CBN [10]. Smoking has also been attributed as a major cause for the occurrence of CBN [12].

Association of CBN with any long-term disease is not known. Patients with CBN do not present with unique clinical features and do not appear to have any difference in outcomes for common forms of infections such as the respiratory infections, between individuals with and without neutropenia from the same ethnic population. Some of the patients with CBN were found to have normal bone marrow morphology as well as other leukocyte counts (lymphocytes, basophils, eosinophils, and monocytes). In some instances CBN is associated with several clinical manifestations ranging from mild infections to severe disease conditions. In certain random cases patients identified with severe congenital neutropenia were associated with a premalignant condition with an increased risk of clonal hematopoietic diseases such as MDS and leukaemia [13].

Very few studies have been reported on the incidence and the underlying pattern of this disease. Based on our literature review there were no previous report on the prevalence of this disease in the kingdom of Saudi Arabia which has a total population between 25 and 35 million. Considering the previous reports, 10-15% prevalence in the Middle East, this could results in a significant number of subjects in the Kingdom to this benign condition. Thus, in the present study, we tested 100 samples from subjects who came for regular screening and were not treated for any specific disease, at the King Abdulaziz University Hospital in Jeddah, Kingdom of Saudi Arabia. The current study evaluated the frequency of CBN in the normal population samples and a total of 19 different parameters were analyzed. To achieve our objectives, we used flow cytometry to measure cell population data (CPD) as well as cell surface markers to more specifically categorize the peripheral blood lymphocytes on subjects with CBN compared to subjects without CBN. To our knowledge the current study is the first of its type to look into factors associated with CBN in the Middle East population focused on the general population in the Kingdom of Saudi Arabia.

## Methods

Peripheral blood samples were obtained from patients and healthy volunteers (males, 18 - 55 years; females, 18 - 50 years) into dipotassium EDTA-anticoagulant in Vacutainer tubes (Becton Dickinson, Franklin Lakes, NJ, USA) and stored at room temperature. Absolute blood cell counts as well as peripheral blood lymphocyte counts were determined using BD FACS Canto II flow cytometer (San Jose, California, USA) using fluorescent labelled antibodies. In this pilot study 100 samples obtained from normal healthy subjects were analzyed. The subjects enrolled in the study are ethnic to Saudi Arabia and included both Saudi (n = 69) and non-Saudi (n = 31) population. The subjects were randomly selected from those visiting the hospital for routine check-up. There was no bias associated in selection of individuals and the final study group comprised of 89 males and 11 females. The questionnaire collected included history of smoking, acute and chronic infection at different sites, active autoimmune disease, drug therapy, family history and pregnancy if the subject is female, The following parameters namely, history of (1) bacterial infection, (2) viral infection, (3) rheumatic infection, (4) skin infection, (5) diarrhoea, (6) chest infection, (7) ear infection, (8) history of medication, (9) herpes infection, (10) smoking habits, (11) osteoarthritis, (12) pregnancy, (13) Systemic lupus erythematosus (SLE), (14) rheumatoid arthritis (RA), (15) current cancer therapy, (16) vitamin deficiencies, and (17) radiation therapy were included in the study to understand the underlying cause for the cause for CBN.

Cell population data (CPD) was collected for (1) WBCs, (2) neutrophils, (3) lymphocytes, (4) monocytes, (5) eosinophils and (6) basophils. Peripheral blood lymphocyte subsets (CD3, CD3+CD8, CD3+CD4, CD16+CD56 and CD19) were quantified to study the role of T-cells, lymphocytes, B-cells and their association with CBN.

## Statistical analysis

All statistical analyses were performed using the Statistical Package for Social Sciences (SPSS) software version 20.0 (SPSS, Chicago, IL, USA). The risk factors for the incidence of CBN were evaluated by Chi-square test for categorical variables, and Mann-Whitney U test for numerical variables. Correlation between neutrophil counts with other parameters was evaluated by Pearson’s correlation coefficient.

## Ethical approval

The study was approved by the ethical committee at the Centre of Excellence in Genomic Medicine Research (CEGMR), King Abdulaziz University (HA-02-J-003), following the ethical standards and guidelines of the National Ethical Committee of Saudi Arabia. Written informed consent was obtained from all individuals who participated in the current study and all were all adults.

## Results and discussion

The summary characteristics of 100 subjects with various parameters (19 parameters) used for the current study are provided in Table 1. Based on the current study on the subjects (healthy adults) we did not find any significant association between the prevalence of CBN with the 19 parameters on the decrease in the neutrophil count except for the age and nationality. Saudi nationality was found to be more susceptible to this disease condition compared to the non-Saudi population. All patients diagnosed with CBN were found to have low white blood cells differential count except for basophils. Total lymphocytes, eosinophils, and CD19 subset showed significant differences in the subjects with CBN compared to those who had normal neutrophil count (Table 2).

**Table 1:** Table describing the 19 parameters used to ascertain the health status of subjects who enrolled for the current study. Statistical analysis was carried out to determine the significance of low neutrophil count and its association with the 19 parameters described in the table. A *p* value of < 0.05 is considered to be significant. Y = yes; N = no, NA = not applicable.

Number	Parameters	Y/N/NA	p - value
1	Gender (Male Female)	N	***p*- value**

2	Age	V	p = 0.306

3	Nationality (Saudi / non-Saudi)	Y	***p* = 0.001**

4	History of bacterial infection	NA	***p* < 0.050**

5	History of viral infection	NA	NA

6	History of rheumatic infection	N	NA

7	History of Skin infection	N	*p* = 0.403

8	History of diarrhoea	N	*p* = 0.618

9	History of chest infection	N	*p* = 0.508

10	History of ear infection	N	*p* = 0.122

11	History of medication	N	*p* = 0.403

12	History of smoking	N	*p* = 0.440

13	History of arthritis	N	*p* = 0.443

14	Pregnancy	NA	*p* = 0.50S

15	Systemic lupus erythematosus (SLE)	NA	NA

16	Rheumatoid arthritis	NA	NA

17	History of cancer chemotherapy	NA	NA

18	Vitamin deficiencies	N	NA

19	History of radiation therapy	NA	*p* = 0.638

**Table 2:** Study subjects, mean differential values and prevalence of CBCs associated with chronic benign neutropenia in the Kingdom of Saudi Arabia. A *p* value of < 0.05 is considered to be significant.

Males	89		
Females	11		

Saudi	69		

Non-Saudi	31		

	**Mean Age**	***yrs* (range)**	

All	25.11	(18-55)	

Males	25.26	(18-55)	

Females	23.91	(18-50)	

	**(× 10^9^/L)**	**Mean (Range)**	**Association with neutrophil count**

WBCs	(× 10^9^/L)	7.910(2.59-13.90)	***p* = <0.05**

Neutrophils	(× 10^9^/L)	3.350(0.99-8.88)	***p* = <0.05**

Lymphocytes	(× 10^9^/L)	3.240(0.77-5.96)	***p* = <0.05**

Monocytes	(× 10^9^/L)	0.809(0.001-2.19)	***p* = <0.05**

Eosinophiles	(× 10^9^/L)	0.024(0.008-1.49)	***p* = <0.05**

Basophils	(× 10^9^/L)	0.262(0.00-0.957)	*p* = 0.167

CD3	%	70.68(56.29-94.03)	*p* = 0.070

CD3 + CD8	%	28.93 (14.45-52.10>	*p* = 0.062

CD3 + CD4	%	37.96(27.23-50 08)	*p* = 0.590

CD16 + CD56	%	13.64(0.14-31.74)	*p* = 0.857

CD 19	%	13.97(0.07-26.90)	***p* = < 0.05**

Peripheral blood lymphocyte subsets, CD3, CD3+CD8, CD3+CD4, CD16+CD56 showed no correlation with CBN except for CD19. CD3 (T-cells), CD3 + CD8 (lymphocytes), and CD3 + CD4 (T-cells) showed negative correlation with neutrophils. B-lymphocyte antigen (CD19) alone showed significant difference in the expression pattern (r = 0.395; *p* = <0.0001) in healthy subjects when matched against patients with the benign disease condition. The trend in neutrophils count in subjects with CBN generally correlated with (1) WBC (r = 0.802); (2) lymphocytes, (r = 0.301); (3) monocytes, (r = 0.297); (4) eosinophils, (r = 0.221) and (5) basophils (r = 0.143) (Table 3). Neutrophil counts in patients and CD counts were found to be positively correlated with CD19 (r = 0.395) and CD16+CD56 (r = 0.00) and inversely correlated with CD3 + CD4 (r = -0.110), CD3 (r = -0.279) and CD3+CD8 (r = -0.229) (Table 4).

**Table 3:** Correlation analysis describing the association between neutrophils and different blood cell components.

Number	Cell types	Correlation	**p**- **value**
1	WBC	*r* = 0.802**	**0.000**

2	Lymphocytes	*r* = 0.301**	**0.002**

3	Monocytes	*r* = 0.297**	**0.003**

4	Eosnophils	*r* = 0.221*	**0.027**

5	Basophils	*r* = 0.143	0.155

**Table 4:** Correlation analysis describing the association between peripheral blood lymphocyte counts and neutrophils. Numbers indicated in bold indicates significant difference between healthy and patients with CBN condition.

Number	Peripheral blood lymphocyte subsets	Correlation	*p* = value
1	CD3	*r* = -0.279^*^	**0.013**

2	CD3+CD8	*r* = -0.229^*^	**0.042**

3	CD3+CD4	*r* = -0.110	0.337

4	CD16+CD56	*r* = 0.000	0.997

5	CD 19	*r* = 0.395^**^	**0.000**

The incidence of CBN is more common in certain endemic regions across the globe. The real prevalence of this disease is still not fully established. The prevalence of this disease is also on the rise in the Middle East population due to several factors, consanguinity of marriages perhaps being one of the reasons. The current study has shown an increasing trend in the incidence of this disease based on previous reports [11]. Our study has shown a decreasing trend in the neutrophil count in non-symptomatic populations which is associated with a general decrease in the total blood cell count for WBCs, leucocytes, monocoytes, eosinophils and the CD19 subset (B-lymphocyte). This trend could lead to a more chronic disease condition in the patients affected by this condition. Efforts should be initiated to understand the molecular cause for the decrease in the TBC count in this target population.

Benign ethnic neutropenia has been previously reported in American and British population of African descent and has been associated with a regulatory variant in the Duffy Antigen receptor for chemokines gene [15]. Fewer studies have reported the incidence of this condition in Middle Eastern population such as black Bedouin Arabs (Shoenfeld et al., 1998). Chronic benign neutropenia has not been associated with any increased risk of infection and has also been described in other populations around the world including Africans, African Americans and Afro-Caribbean's [10]. The findings from our studies are similar to other earlier reports when the benign condition was matched with known potential causes for observed low neutrophil counts.

Previous studies have reported that CBN in an Arab tribe is inherited as an autosomal dominant trait with an ANC < 2 x 10^9^/L as a case of neutropenia (Shoenfeld et al., 2010). One of the reasons for a high incidence in Arab population could be due to founder gene effects as well as the result of high percentages of consanguineous marriages among the population. The geographical proximity of Saudi Arabia with the North African countries could also be associated with the migration of those people to the peninsula thus increasing the neutropenia cases in the country.

Previous reports have shown the occurrence or degree of disease severity in categories associated with those of CBN in the Arab population [4,15]. The current study shows that the prevalence of CBN is as high as 20% in subjects with a mean of 1.48 (range 0.99–1.95) (×10^9^cells/L), without any significant differences between the sexes. Ethnicity is reported to contribute to the neutrophils count and hence our studies showed significant differences between Saudi and non-Saudi population. We found concordant decrease in the cell counts for neutrophils and monocytes that indicate a common mechanism may be involved in the regulation of the common progenitor from which these cells arise, leading to CBN in the general population.

Common causes of neutropenia were excluded in this study. Our findings support previous reports on neutropenia in the Arab peninsula. In Saudi Arabia, the incidence of neutropenia is reported mostly on people of Sudanese origin. We observed that 20% of the random samples selected had CBN. This supports the previous reports of the exceptionally higher prevalence of CBN in this part of the world.

Few studies have looked into the cause of congenital neutropenia at the genomic level. Recent report by Xia et al., (2009) [16] showed prevalence of mutations in ELANE, GFI1, HAX1, SBDS, WAS and G6PC3 in patients with severe congenital neutropenia. Among the different mutation studied for neutropenia, mutations associated with ELANE gene were found to be more common [16]. Mutations in B-lymphocyte antigen (CD19) associated with the WASP gene is one potential genetic cause for neutropenia [17,18]. Plasma chemokine levels, particularly CXCL8 and CCL2, were previously reported to be higher in patients who were homozygous for the null allele when compared to patients who were heterozygous following lipopolysaccharide stimulation of their whole blood *in vitro*[19]. The evidence pointed at a locus on chromosome *1q22* which could be responsible for this condition. Later it was found that this polymorphism mapped in this population is within the Duffy antigen receptor chemokine (DARC) gene. This gene encodes the Duffy antigen, a chemokine receptor found on red blood cells [20].

**Figure 1:**
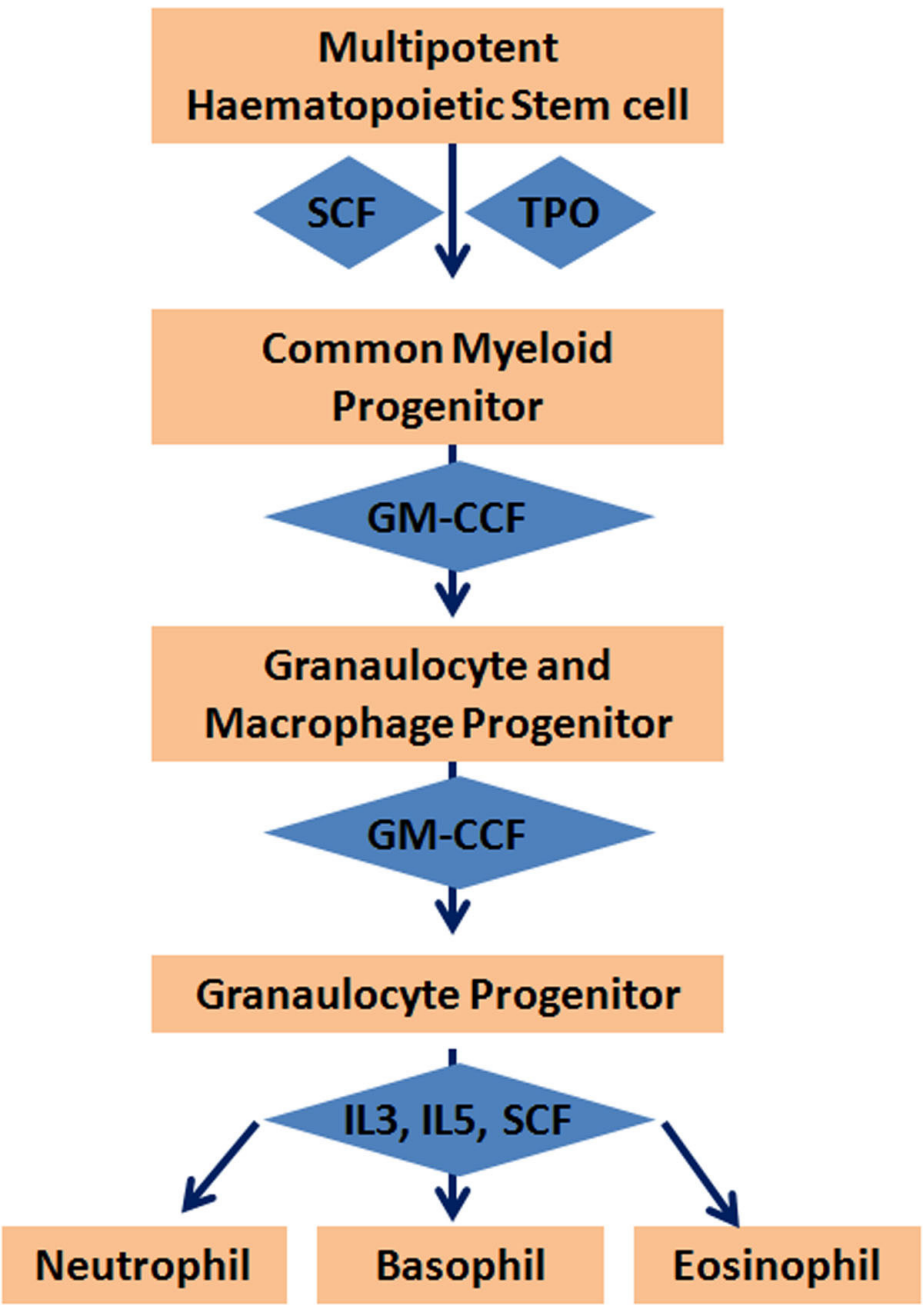
Multipotent haematopoietic stem cell pathway associated with the production of different blood cell lineages. The highlighted gene in colour is associated with neutrophil production (SCF, TPO, G-CSF, IL-3, IL-5). These genes need to be investigated further to screen for any mutations that could result in an aberrant gene which could inhibit the synthesis of neutrophils leading to a lower neutrophil count. EPO (erythropoietin - cytokine), G-CSF (colony stimulating factor 3 (granulocyte) - cytokine), GM-CSF (colony stimulating factor 2 (granulocyte-macrophage) – cytokine), IL-15 (interleukin 15 – cytokine), IL-2 interleukin 2 – (cytokine), IL-3 interleukin 3 – (cytokine), IL-4 (interleukin 4 – (cytokine), IL-5 (IL-5 interleukin 5 – (cytokine), IL-7 (interleukin 7 – (cytokine), M-CSF colony stimulating factor 1 (macrophage) - cytokine, SCF (KIT ligand) – growth factor, TPO (thrombopoietin) – cytokine.

Potential cause for the reduction of neutrophils could be attributed to genetic factors especially genes associated with the multipotent haematopoietic stem cell pathway (Fig. 1). Multipotent haematopoietic stem cell pathway is associated with the production of different blood cell lineages including the lymphocyte subsets. Genes associated with the neutrophils include SCF (KIT ligand) growth factor, TPO (cytokine), G-CSF (growth factor), IL-3 (interleukin), IL-5 (interleukin) (Table 5). Aberrant mutations in these genes could result in the inhibition of synthesis of neutrophils leading to a lower neutrophil as well as other cell counts. It has been found that TPO gene enhances neutrophil production by bone marrow hematopoietic progenitors with the aid of stem cell factor in congenital neutropenia [21].

**Table 5:** Genes associated with haematopoiesis and that could play a role in neutrophil count in chronic benign neutropenia.

Number	Entrez Gene Name	Symbol	Family
1	Erytliropoietin	EPO	cytokine

2	Colony slimulaling factor 3 (granulocyte)	G-CSF	cytokine

3	Colony slinuilaling factor 2 (granulocyte-macrophage)	GM-CSF	cytokine

4	Interleukin 15	IL-15	c\1okine

5	Interleukin 2	IL-2	cytokine

6	Interleukin 3 (colony-stimulating factor, multiple)	IL-3	other

7	Interleukin 4	IL-4	cytokine

8	Interleukin 5 (colony-stimulating factor, eosinophil)	IL-5	cytokine

v	Interleukin 7	II.-7	cytokine

10	Colony stimulating factor 1 (macrophage)	M-CSF	cytokine

11	KIT ligand	SCF	growth factor

12	Thrombopoietin	TPO	cytokine

Granulocyte colony-stimulating factor (G-CSF) plays a key role in the hematopoietic stem cell pathway. Severe congenital neutropenia was to found to be associated with abnormal growth and differentiation of myeloid progenitors to granulocyte colony-stimulating factor (G-CSF). However they showed normal response to G-CSF plus stem cell factor [22]. Similarly Interleukins (IL-3, IL-4 and IL-5) play a key role in differentiation and proliferation of hematopoietic cells [23]. Identification mutations in genes across affected families in endemic countries could lead to the identification of the cause for the CBN in Middle East Countries.

## Conclusions

Our studies show the mean neutrophil count in Saudi populations affected with CBN is even lower than those reported in the general Arab population. The concurrent decrease in other peripheral blood mononuclear cell counts is reported for the first time and this could be associated with a latent stage of even more severe conditions associated with the CBN. Though these patients do not present with any visible disease conditions, a long-term plan should be put in place to monitor the health conditions of these individuals. In the future, it is recommended that a more extensive screening should take place in the general population to dissect the genetic basis of CBN in the Saudi Arabia population and to determine if this is similar to other populations that also have an increase in CBN.

## List of abbreviations

CBN: Chronic benign neutropenia; CD19: Cell differentiation antigen 19; CPD: Cell population data; FACS: Fluorescence activated cell sorting; G-CSF: Granulocyte colony-stimulating factor; IL: Interleukins; MDS: Myelodysplastic syndromes; SCN: Severe chronic neutropenia; WBC: White blood cells

## Competing interests

The authors declare no competing interests associated with this publication.

## Contributions from different authors

MAG designed and performed the research study; MD, AG, EA and RJ completed the questionnaires and carried out the experiments; KN, SL and KG performed the data analysis, manuscript preparation and editing; FAS helped in review of manuscript and haematological parameters; AC, AA and MQ contributed to the reagents/kits and facilities.

## Declarations

Publication charges for this article have been funded by the Center of Excellence in Genomic Medicine Research (CEGMR), King Abdulaziz University, Jeddah, 21589, Kingdom of Saudi Arabia.

This article has been published as part of *BMC Proceedings* Volume 9 Supplement 2, 2015: Selected article from the 2nd International Genomic Medical Conference (IGMC 2013): Proceedings. The full contents of the supplement are available online at http://www.biomedcentral.com/bmcproc/supplements/9/S2
